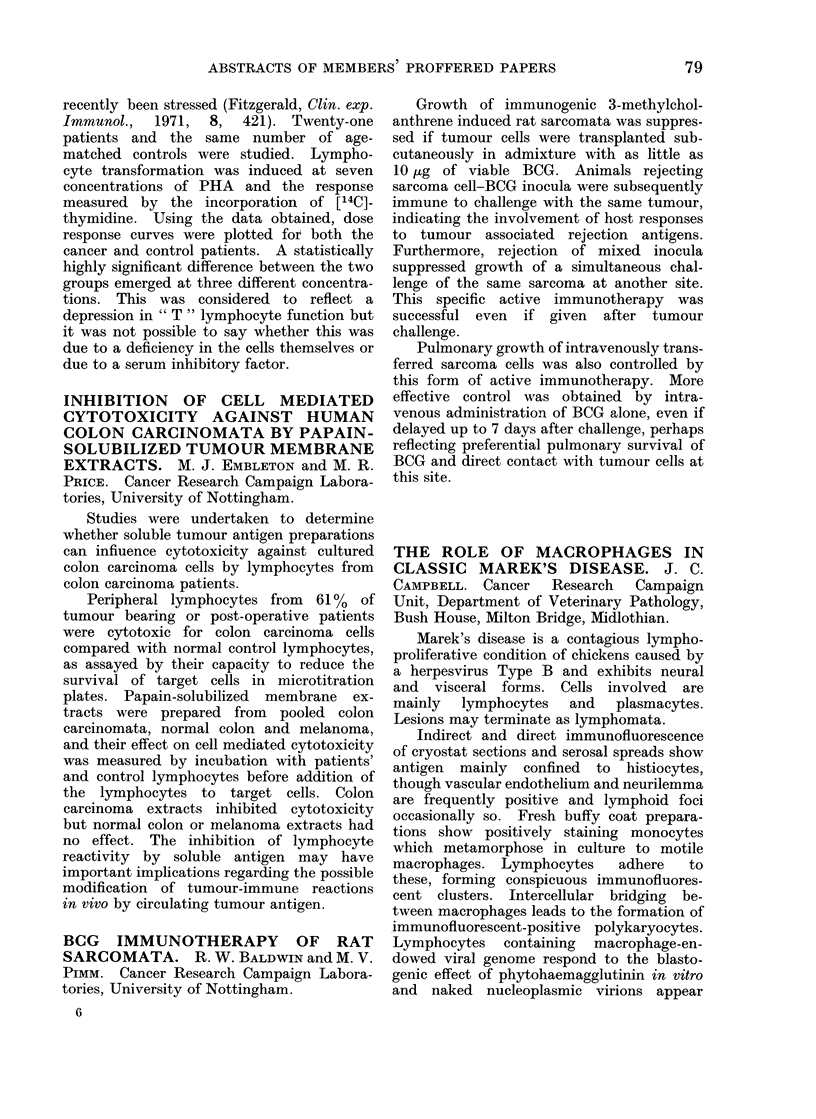# BCG immunotherapy of rat sarcomata.

**DOI:** 10.1038/bjc.1973.84

**Published:** 1973-07

**Authors:** R. W. Baldwin, M. V. Pimm


**BCG IMMUNOTHERAPY OF RAT SARCOMATA.** R. W. Baldwin and M. V. Pimm. Cancer Research Campaign Laboratories, University of Nottingham.

Growth of immunogenic 3-methylcholanthrene induced rat sarcomata was suppressed if tumour cells were transplanted subcutaneously in admixture with as little as 10 $\mu$g of viable BCG. Animals rejecting sarcoma cell–BCG inocula were subsequently immune to challenge with the same tumour, indicating the involvement of host responses to tumour associated rejection antigens. Furthermore, rejection of mixed inocula suppressed growth of a simultaneous challenge of the same sarcoma at another site. This specific active immunotherapy was successful even if given after tumour challenge.

Pulmonary growth of intravenously transferred sarcoma cells was also controlled by this form of active immunotherapy. More effective control was obtained by intravenous administration of BCG alone, even if delayed up to 7 days after challenge, perhaps reflecting preferential pulmonary survival of BCG and direct contact with tumour cells at this site.